# When does attrition lead to biased estimates of alcohol consumption? Bias analysis for loss to follow‐up in 30 longitudinal cohorts

**DOI:** 10.1002/mpr.1842

**Published:** 2020-07-13

**Authors:** Katherine M. Keyes, Justin Jager, Jonathan Platt, Caroline Rutherford, Megan E. Patrick, Deborah D. Kloska, John Schulenberg

**Affiliations:** ^1^ Department of Epidemiology, Mailman School of Public Health Columbia University New York New York USA; ^2^ School of Social and Family Dynamics Arizona State University Tempe Arizona USA; ^3^ Institute for Translational Research in Children's Mental Health University of Minnesota Minneapolis Minnesota USA; ^4^ Institute of Child Development University of Minnesota Minneapolis Minnesota USA; ^5^ Institute for Social Research University of Michigan Ann Arbor Michigan USA; ^6^ Department of Psychology University of Michigan Ann Arbor Michigan USA

**Keywords:** adolescents, alcohol, attrition

## Abstract

**Objectives:**

Survey nonresponse has increased across decades, making the amount of attrition a focus in generating inferences from longitudinal data. Use of inverse probability weights [IPWs] and other statistical approaches are common, but residual bias remains a threat. Quantitative bias analysis for nonrandom attrition as an adjunct to IPW may yield more robust inference.

**Methods:**

Data were drawn from the Monitoring the Future panel studies [twelfth grade, base‐year: 1976–2005; age 29/30 follow‐up: 1987–2017, *N* = 73,298]. We then applied IPW imputation in increasing percentages, assuming varying risk differences [RDs] among nonresponders. Measurements included past‐two‐week binge drinking at base‐year and every follow‐up. Demographic and other correlates of binge drinking contributed to IPW estimation.

**Results:**

Attrition increased: 31.14%, base‐year 1976; 61.33%, base‐year 2005. The magnitude of bias depended not on attrition rate but on prevalence of binge drinking and RD among nonrespondents. The probable range of binge drinking among nonresponders was 12–45%. In every scenario, base‐year and follow‐up binge drinking were associated. The likely range of true RDs was 0.14 [95% CI: 0.11–0.17] to 0.28 [95% CI: 0.25–0.31].

**Conclusions:**

When attrition is present, the amount of attrition alone is insufficient to understand contribution to effect estimates. We recommend including bias analysis in longitudinal analyses.

## 1 INTRODUCTION

One of the greatest threats to continued advances in the understanding of etiology of substance use across the life span concerns attrition from longitudinal studies. Survey nonresponse and longitudinal study attrition rates have increased substantially in recent decades in the United States (Galea & Tracy, 2007; Keeter, Kennedy, Dimock, Best, & Craighill, 2006; Tolonen et al., 2006). Increases in other countries have been even greater (Johnson & Wislar, 2012; Tolonen et al., 2006). Attrition rates in large‐scale longitudinal studies in the United States depend on the modality of data collection [e.g., mail, cellphone, and web] (Patrick et al., 2017) and range, for example, from 25% in the National Longitudinal Survey of Adolescent to Adult Health (Harris et al., 2019), to 40–55% in the National Longitudinal Study of Youth (Aughinbaugh & Gardecki, 2007), 40–60% in the Population Assessment of Tobacco and Health study (Hyland et al., 2017), 44% in the Panel Study of Income Dynamics (McGonagle, Schoeni, Sastry, & Freedman, 2013), and 30–50% in other large‐scale longitudinal studies in the United States and other countries (Bureau of Labor Statistics, 2020a, 2020b).

Attrition and nonresponse do not necessarily result in biased associations between exposures and outcomes with respect to the target population if random (Groves & Peytcheva, 2008). However, even studies with low attrition can generate biased associations if existing attrition is nonrandom; that is, when attrition is related to examined exposures and outcomes and selection out of the cohort (Greenland, 1977; Gustavson, Von Soest, Karevold, & Roysamb, 2012; McCabe & West, 2016), depending on assumptions regarding the unobservable causal structure of attrition and the measure of association (Daniel, Kenward, Cousens, & De Stavola, 2012; Howe, Cole, Lau, Napravnik, & Eron, 2016; Westreich, 2012). The increases in overall attrition and nonresponse across historical time in many longitudinal studies have brought these issues to the forefront of attention in generating appropriate inferences in the presence of high rates of attrition.

Well‐developed methods for adjusting for nonresponse and attrition are in widespread use; inverse probability weight [IPW] (Little & Rubin, 2002; Rubin, 1976) is commonly applied, although not the only approach that could be used. For IPWs to eliminate bias, however, the model to estimate weights needs to be correctly specified, including no omitted variables contributing to attrition that are causes of study exposures and outcomes (Hogan & Lancaster, 2004; Robins, Hernán, & Brumback, 2000). One way to additionally bound the potential for bias due to attrition when it is unclear whether assumptions are met is through quantitative bias analysis (Greenland & Lash, 2008; Lash et al., 2014; Lash, Fox, & Fink, 2009). For attrition, quantitative bias analysis can augment weighting algorithms to provide more information regarding the potential for residual bias. Given that the long‐term outcomes among those lost to follow‐up are unknown and that methods to adjust longitudinal studies for attrition require assumptions, bias analysis can provide a transparent assessment of the potential for incorrect inference after adjustment for known causes of attrition.

Assessing the potential for increasing bias due to attrition in recent historical time is especially important for examining effects with early onset and potentially long duration, such as the consequences of adolescent alcohol use. Heavy and prolonged alcohol use is a leading contributor to global morbidity (Rehm & Shield, 2013; Whiteford et al., 2013; World Health Organisation, 2014) and is most likely to begin during adolescence (Patrick & Schulenberg, 2016). Assessing the extent to which adolescents increase, decrease, or stably engage in binge drinking as they become adults informs developmental science and public health (Schulenberg & Maggs, 2002). Longitudinal data are critical to assessment of binge drinking stability during the transition to adulthood. Yet, substance use, including alcohol use, is a key factor that predicts attrition out of longitudinal studies (McCoy et al., 2009; Osler, Kriegbaum, Christensen, Holstein, & Nybo Andersen, 2008; Zhao, Stockwell, & Macdonald, 2009), creating difficulties in inference.

The goal of the present article is to provide a guide and framework to the field for conducting quantitative bias analysis in longitudinal cohorts for the purpose of understanding life course stability of substance use. We used Monitoring the Future [MTF] data from 30 sequential cohorts followed from age 18 [assessed in twelfth grade in years 1976–2005] to age 29/30 [assessed in years 1987/1988 to 2016/2017]. The design and measures have remained nearly constant through the almost four decades, but attrition has increased across historical time, as in many studies. We conducted quantitative bias analysis in these cohorts by imputing data on binge drinking at age 29/30 among nonresponders. These imputations made a series of assumptions that allow us to generate the range of possible true associations between age 18 and age 29/30 binge drinking if we had followed the entire cohort throughout the study period. First, we generated imputations that tested whether we can completely eliminate the relationship between adolescent to adult binge drinking under any assumed level of binge drinking among nonrespondents. Second, we estimated the most plausible range of likely prevalence of binge drinking among nonrespondents to provide a plausible range of true associations.

## 2 METHODS

### 2.1 Sample

The Monitoring the Future study includes nationally representative samples of approximately 15,000 high school seniors [twelfth grade] surveyed annually since 1976 (Miech et al., 2019). From the annual survey, ~2,450 students are randomly selected for longitudinal follow‐up, with oversampling for students who report drug use (Schulenberg et al., 2019). These respondents begin follow‐up assessments either 1 [modal age 19] or 2 [modal age 20] years later and are followed every 2 years thereafter (Miech et al., 2019; Schulenberg et al., 2019). We limited analysis to cohorts who had the opportunity to be followed through follow‐up 6 [age 29/30] by 2017. The total sample size at modal age 18 was 73,298. An Institutional Review Board of University of Michigan approved the study.

### 2.2 Measures

#### 2.2.1 Binge drinking

At baseline [age 18] and age 29/30, respondents were asked to think back over the past 2 weeks and answer “How many times have you had five or more drinks in a row?” We examined binge drinking as any versus none.

#### 2.2.2 Covariates included in inverse probability of attrition weights [IPW]

Stabilized weights were estimated based on predicted risk of attrition, conditional on correlates of attrition over the study follow‐up. These included: binge drinking status at each wave [yes/no], study year, gender, race/ethnicity, highest education level of mother and father as reported by the adolescent, high school grade point average, and a survey weight adjusting for oversampling of some groups. Supporting Information Table S1 provides an overview of the study covariates, and their association with attrition.

### 2.3 Statistical analysis

First, we estimated the unadjusted risk difference [RD] for age 29/30 binge drinking between those who engaged in baseline binge drinking versus those who did not, with no adjustment for attrition. RD and 95% confidence intervals were estimated from generalized linear models using a log‐binomial link. Units of the RD are per 100 persons observed.

Second, we reestimated the RD of age 29/30 binge drinking, weighting estimates based on the inverse of their probability of nonresponse [attrition] at each wave of follow‐up.

Third, we applied bias analysis to the assessment of the relationship between age 18 binge drinking and age 29/30 binge drinking. We simulated a range of 0–100% of those lost to follow‐up engaging in age 29/30 binge drinking, at the same proportion among those who were binge drinking at baseline. This assumption fixes the unobserved RD of adolescent to adult binge drinking to 0 among those lost to follow‐up. This analysis allows us to examine the scenario in which *no* students who were lost to follow‐up engaged in age 29/30 binge drinking, or alternatively, that *all* students who were lost to follow‐up engaged in age 29/30 binge drinking. Because losses to follow‐up increased over historical time, the number of respondents who were imputed increased across historical time.

However, it is unlikely that the unobserved RD among those lost to follow‐up is zero, or that 0% or 100% of those lost to follow‐up were binge drinking at age 29/30. The question, then, becomes, “What is a valid estimate for the unobserved RD?” For the fourth step, we simulated three scenarios. (a) That the unobserved RD among those lost to follow‐up is equal to the observed RD among those not lost to follow‐up, within each cohort group; (b) that the unobserved RD among those lost to follow‐up was *lower* than the observed RD, by 0.1 points. The rationale for this assumption is that adolescents who have a greater prevalence of risk factors for adult binge drinking, other than adolescent binge drinking, may be more likely to be lost to follow‐up, which would serve as competing risks; and (c) that the unobserved RD among those lost to follow‐up was *higher* than the observed RD among those not lost to follow‐up, again by 0.1 points. The rationale for this assumption was that binge drinking at baseline may be a particularly strong risk factor for study drop‐out, and for adult binge drinking, and thus may be a stronger risk factor among those lost to follow‐up compared to those observed.

Finally, in a fifth step, we attempted to estimate the most plausible range of likely prevalences of binge drinking as if there had been no attrition. We assumed those who have a high probability of attrition, but did not drop out, are our best estimate of the prevalence of age 29/30 binge drinking among those who did drop out. We created quartiles of sequentially increasing probability of attrition [e.g., 0–24% and 25–49%] among those who remained in the study, using the same predicted attrition risk model used to calculate IPWs. Within each stratum, we estimated age 29/30 binge drinking for those who were not lost to follow‐up, and also estimated the RD of age 29/30 binge drinking based on baseline binge drinking status.

All analyses were conducted using SAS [version 9.4], and figures were created with the ggplot2 package in R (Wickham, 2016). Code for all analyses is available in the Supporting Information.

## 3 RESULTS

**FIGURE 1:**
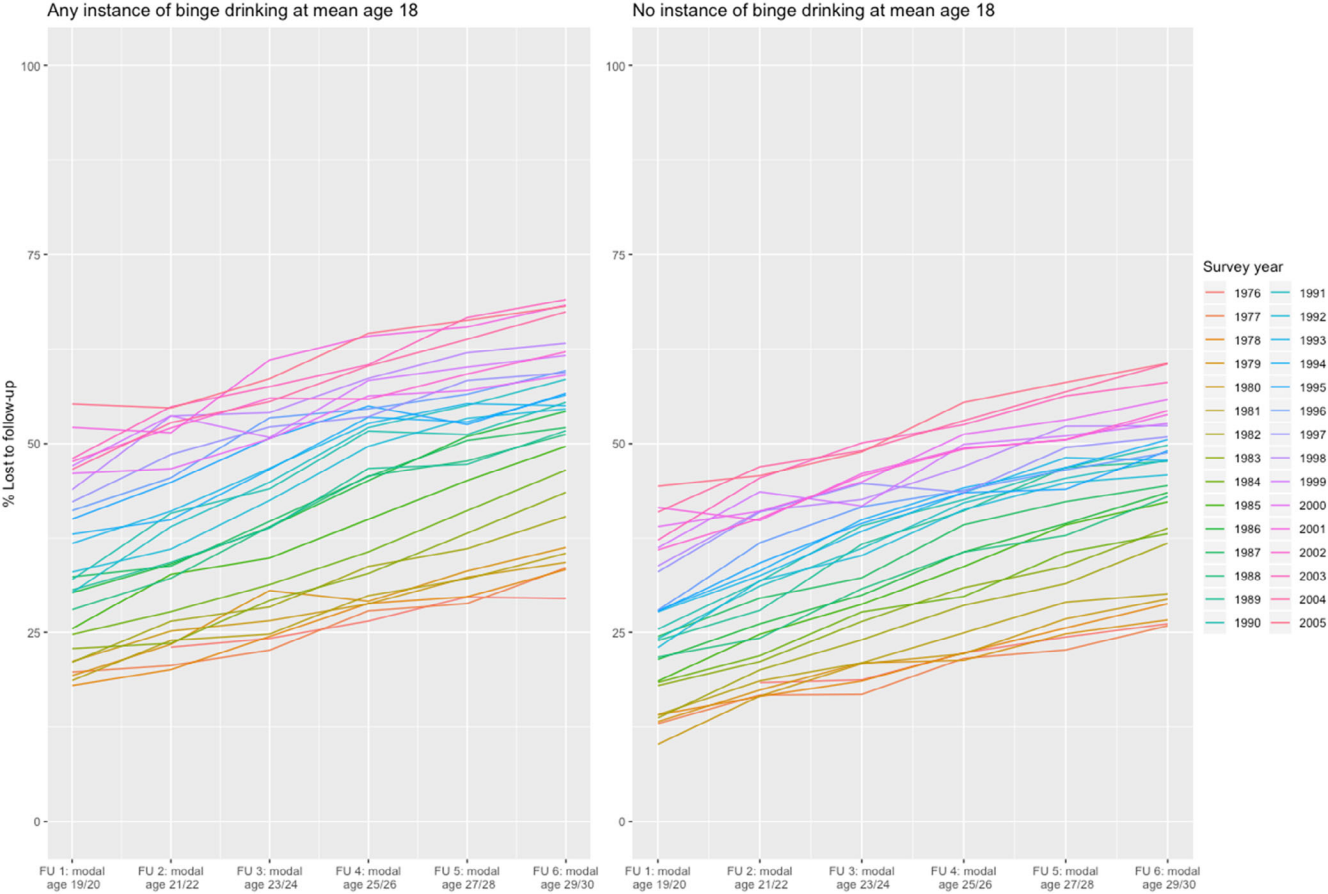
Attrition percentages in MTF from baseline 1976 through baseline 2005, stratified by binge drinking status at age 18. MTF, Monitoring the Future

Across all cohorts, and without incorporating any attrition weights or adjustment, 37.2% of adolescents reported binge drinking at age 18, with declines across historical time, from 48.6% in 1976–1980 to 34.1% in 2001–2005. Figure 1 provides an overview of attrition in MTF participants. Each line represents the attrition percentage by cohort, at baseline and at age 29/30. Across cohorts, the largest amount of attrition occurred between age 18 and age 19/20; indeed, of the total attrition across historical and developmental time, 45.7% occurred between baseline and follow‐up 1, leaving 54.3% of the total attrition occurring from age 19/20 to 29/30. Attrition increased across more recently followed historical cohorts. Although attrition increased across time, the association between baseline binge drinking and attrition did not vary [cohort group by baseline binge drinking interaction was tested; the interaction parameter was 0.062 (SE = 0.133), *p* = .64].

### 3.1 Association between baseline and age 29/30 binge drinking with no attrition adjustment

Table 1 shows the percent lost to follow‐up by 5‐year cohort groups, as well as the RD for the association between baseline and age 29/30 binge drinking status with no adjustment for attrition. Without attrition adjustment, the RD ranged from 0.24 [95% CI 0.21–0.27] among those in the 2001–2005 cohort group [i.e., those who were 18 years old in 2001–2005] to 0.30 [95% CI 0.29–0.33] among those in the 1991–1995 cohort group.

**TABLE 1:** The risk difference association between age 18 and age 29/30 binge drinking status, by 5‐year cohort groups, with no adjustment for attrition, IPWs adjustments, and change between the two

Cohort group	% lost to follow‐up by age 29/30	No adjustment for attrition	IPW for measured predictors of attrition	Change in RD between no IPW and IPW
		RD [95% CI]	RD [95% CI]	
1976–1980	31.14	0.27 [0.25, 0.29]	0.14 [0.12, 0.16]	−0.13
1981–1985	40.65	0.28 [0.26, 0.30]	0.23 [0.21, 0.25]	−0.05
1986–1990	49.46	0.26 [0.24, 0.29]	0.18 [0.16, 0.21]	−0.08
1991–1995	51.61	0.30 [0.28, 0.33]	0.24 [0.22, 0.27]	−0.06
1996–2000	55.92	0.28 [0.26, 0.31]	0.15 [0.12, 0.18]	−0.13
2001–2005	61.33	0.24 [0.21, 0.27]	0.08 [0.05, 0.11]	−0.16

IPWs were estimated as the predicted probability of attrition using a generalized linear model adjusted for baseline binge drinking status, gender, race/ethnicity, mother and father with a college degree, high school grade point average, and drug strata weight [see Supporting Information Table S1 for additional information].

Abbreviations: IPWs, inverse probability weights; RD, risk difference.

### 3.2 Association between baseline and age 29/30 binge drinking with IPW attrition adjustment

Also shown in Table 1, estimates of RD for the association between baseline and age 29/30 binge drinking declined when IPW is applied, with the decline ranging from 66.7% for the 2001–2005 cohort group [from the RDs in Table 1, (0.24–0.08)/0.24] to 17.9% for 1981–1985 cohort group [from the RDs in Table 1, (0.28–0.23)/0.28], at an average decline in the RD of 38.3%. The overall accuracy of the predicted attrition model was moderate [area under the receiver operating characteristic curve = 0.59].

### 3.3 Association between baseline and age 29/30 binge drinking with IPW attrition adjustment and bias analysis: varying prevalence of binge drinking among those lost to follow‐up, RD fixed at 0

**FIGURE 2:**
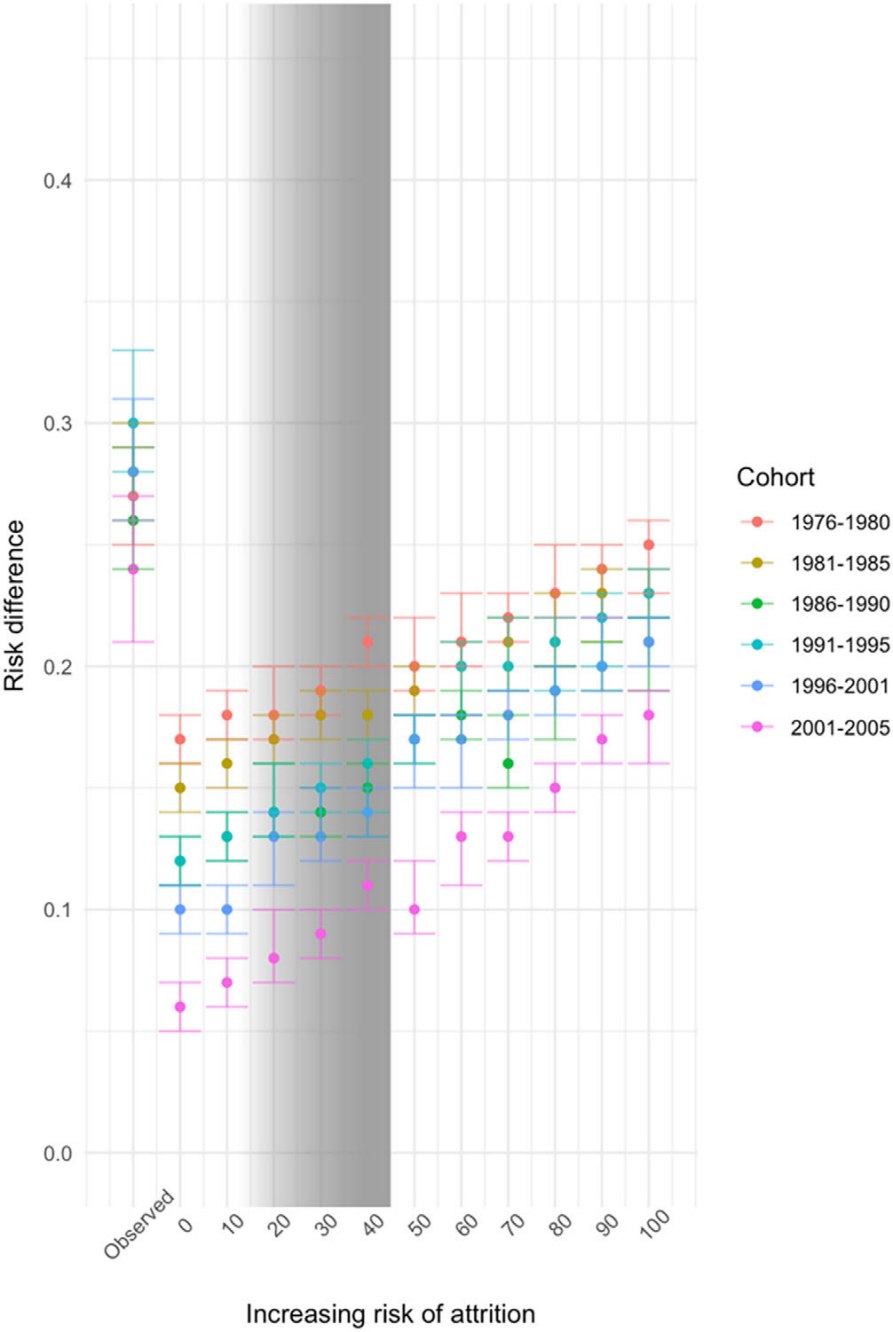
Bias analysis for risk differences with imputation of a range of possible age 29/30 binge drinking statuses among those lost to follow‐up by age 29/30, nondifferentially by baseline binge drinking status [gray bars indicate the probable range of effect sizes with full follow‐up]

Figure 2 includes the RD for the association between baseline and age 29/30 drinking [with IPW attrition adjustment] as a function of the bias analysis in which we iteratively increased by 10 percentage points the imputed proportion of age 29/30 binge drinking that we would have observed if those lost to follow‐up had been observed, assuming that the RD among those lost to follow‐up is 0. All imputed models indicated a lower estimate of RD than what was observed in the IPW‐adjusted estimates of RD [listed in first column in Figure 2, as well as in Table 1]. Thus, under these assumptions, the observed RD for the association is *overestimated* in all scenarios; however there is no scenario in which the relationship between baseline and follow‐up binge drinking is null.

The lowest estimated RD between baseline and age 29/30 binge drinking is under the assumption that no respondent [0%] lost to follow‐up engaged in binge drinking among those in the most recent cohorts [age 18 between 2001 and 2005], in which attrition was highest. As the proportion of binge drinking imputed among those lost to follow‐up increases, so too does the estimated magnitude of RD for the association between baseline and age 29/30 binge drinking. The strongest estimated RD in the imputation models assuming that 100% of those lost to follow‐up would have reported binge drinking was among those in the 1976–1980 cohort, in which attrition was lowest. Of note, the magnitude of RD for the association between baseline and age 29/30 binge drinking generally declines in these simulations among more recent cohort groups, across all range of assumptions about the magnitude of binge drinking among those lost to follow‐up.

### 3.4 Association between baseline and age 29/30 binge drinking with IPW attrition adjustment and bias analysis: varying prevalence of binge drinking and varying RD among those lost to follow‐up

The magnitude of the differences in RDs remains relatively stable across the amount of attrition across cohorts; variation in the magnitude of the RDs is driven by assumptions about increases in the outcome among those lost to follow‐up as well as the association with exposure.

**FIGURE 3:**
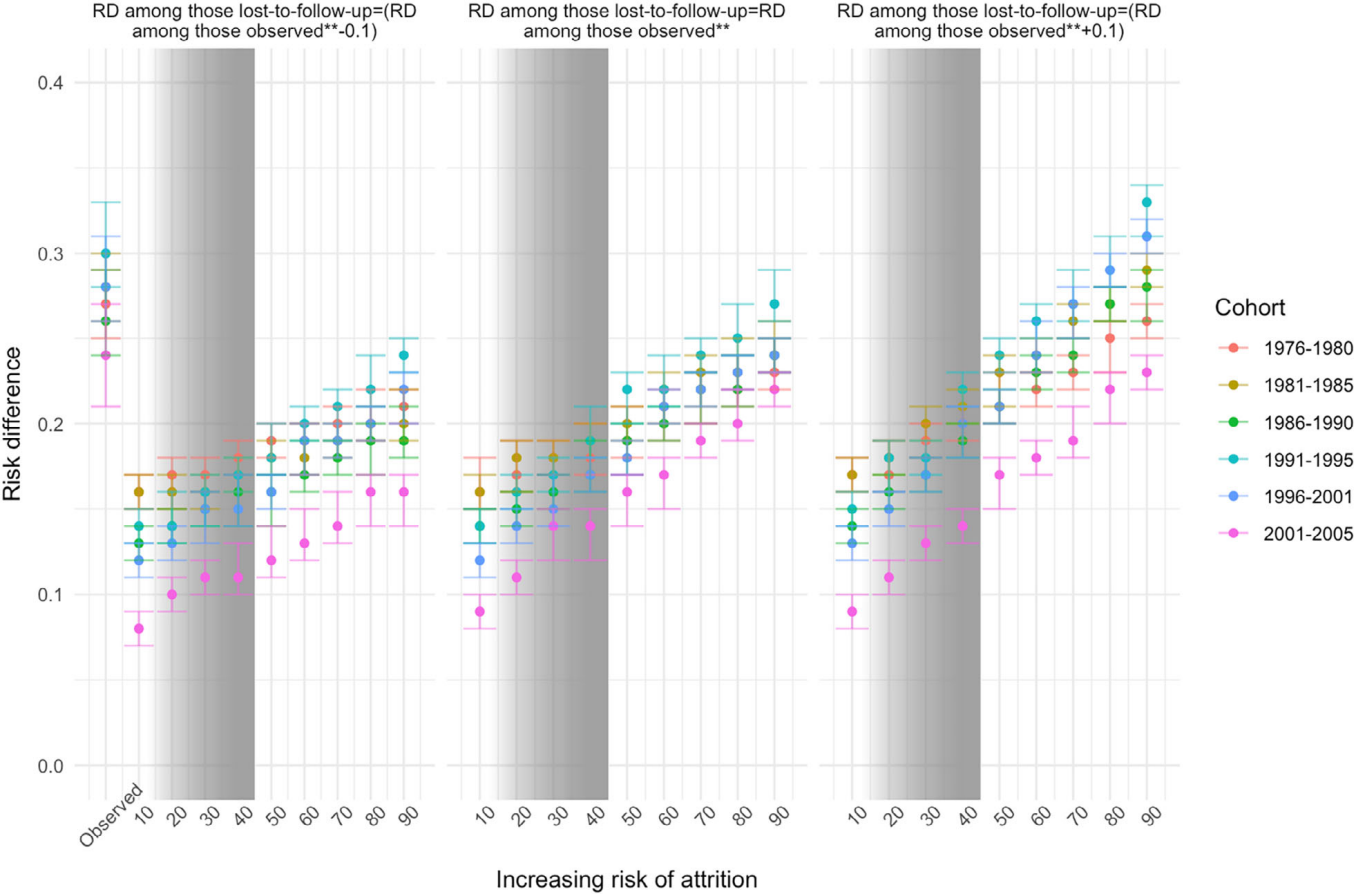
Bias analysis for RDs with imputation of a range of possible age 29/30 binge drinking statuses among those lost to follow‐up by age 29/30 differentially by twelfth grade binge drinking status, fixing the RD among those lost to follow‐up as equal to the observed RD [center panel], less than observed RD [left panel], and greater than observed RD [right panel]. RD, risk difference

Figure 3 presents estimates of a series of RDs with imputation of binge drinking status among those lost to follow‐up under different scenarios in which the magnitude of RD among those lost to follow‐up now depends on three factors: the proportion of attrition, the proportion of binge drinking among those lost to follow‐up, and the association with baseline binge drinking.

When assuming that the unobserved RD among those lost to follow‐up is equal to the observed RD, the effect estimates are always smaller than the observed. The RD varied as a function of cohort, given that different percentages of individuals are lost to follow‐up across time. Generally, decreasing the RD assumed among those lost to follow‐up decreased the overall RD [e.g., the left panel]. Increasing the RD assumed among those lost to follow‐up increased the overall RD [e.g., the right panel]. That is, the more those lost to follow‐up are homogeneous with respect to risk for binge drinking, the lower the overall association would have been if those individuals were retained in the sample. Indeed, the only scenario in which the “true” RD would be greater than the observed RD is one in which the RD between baseline and follow‐up binge drinking was assumed to be higher than observed.

### 3.5 Evaluating the likely range of effects sizes that would have been observed with complete follow‐up

Although the true association between baseline and age 29/30 binge drinking is not knowable, we can provide some bounds on what it likely would have been by estimating the extent of binge drinking that would have been observed among those who were not followed up. In Supporting Information Table S2, we calculated the observed risk of binge drinking at age 29/30 for each quartile of attrition risk and the RD of binge drinking at age 29/30 by baseline binge status. Those with the highest predicted risk of attrition [10–36%] were 1.39 times more likely to binge drink at age 29/30 than those at the lowest predicted risk [95% CI 1.36, 1.40]. Among those with the highest risk of attrition who reported binge drinking at baseline, the RD of age 29/30 binge drinking was 0.28 [95% = 0.25, 0.31]. Using these estimates, we estimated the predicted probabilities of binge drinking among those lost to follow‐up as between 12.35 and 45.87%. Under this range, the true RD is likely between 0.14 [0.11, 0.17] and 0.28 [0.25, 0.31], when the RD among those lost to follow‐up is assumed to be equal to the observed. We inserted gray shading into Figures 2 and 3 that cover the range [i.e., 12.35 to 45.87] of our estimate of the probable relationship between baseline and age 29/30 binge drinking had we observed all cohort members.

## 4 DISCUSSION

The present study used a long‐standing multicohort panel study with variation in the amount of attrition across historical time to address the potential for bias in estimating stability of drinking during the transition to adulthood. The magnitude of attrition alone was insufficient for drawing conclusions about the presence of bias; instead, such conclusions depend on the proportion of the outcome [i.e., age 29/30 binge drinking] among those lost to follow‐up, as well as the RD between baseline exposure and the outcome among those lost to follow‐up. We find that under most scenarios, observed estimates, even with IPW, likely overestimate the association between adolescent and adult binge drinking. However, our estimate of the likely range of effect sizes indicates that age 18 binge drinking is strongly related to follow‐up binge drinking even when accounting for attrition. Importantly, there were no scenarios in which the estimated association between baseline and follow‐up binge drinking was close to zero, underscoring that longitudinal panel data remain essential epidemiological designs even when attrition is high.

These results should be reassuring for longitudinal panel studies, in that while the exact magnitude of the relationship between adolescent substance use and adult outcomes may be affected by differential attrition, the conclusions about the impact of adolescent substance use, at least in the present study, are not. Rather than dismissing studies with high levels of attrition, we advocate that scholars not only consider IPW for known attrition predictors, but also include quantitative bias analysis to further bound the potential for unobserved attrition predictors to influence results. To the extent that conclusions are sensitive to bias, more caution should be taken in inference and recommendations based on results. Indeed, these results indicate that studies even with very high rates of follow‐up are not immune to bias, depending on how the attrition that does occur is associated with study exposures and outcomes. Thus, attrition rates alone are not sufficient indicators of study rigor.

The types of bias that we have described here are often considered selection bias, represented in graphical diagrams as a set of observed or unobserved factors that influence both the outcome of interest as well as selection out of the cohort (Greenland, 1977; Howe et al., 2016; Mansournia, Higgins, Sterne, & Hernán, 2017; Pizzi et al., 2012). The observed cohort, then, becomes a subset of the potential study population, in essence stratifying the analytic cohort on the condition of observed follow‐up. This stratification may result in collider bias [bias that arises due to conditioning or controlling for a common consequent of exposure and outcome] as well as residual confounding (Westreich, 2012), and as we demonstrate in the present analysis, result in observed estimates that may over or under estimate the unobserved true association. However, common criticisms of longitudinal studies tend to focus on the amount of attrition, rather than the magnitude of the association between exposure and outcome among those lost to follow‐up. Indeed, the principle that the amount of attrition matters less than the association with exposures underlies, in part, the interpretation of cumulative incidence case control studies as estimators of risk (Westreich, 2012).

We demonstrate that an IPW for adjusting estimates due to known variables associated with attrition generally attends to some degree of bias, getting closer to a potential “true” effect. Indeed, IPW in the present study reduced the magnitude of estimates by approximately 10%. However, IPW alone may not be sufficient to completely account for differential attrition, especially when the underlying model to predict attrition exhibits suboptimal accuracy. Including bias analysis in ongoing cohort studies may be a useful adjunct to existing methods to increase confidence in the validity of results. Prior studies that elucidate the impact of selection bias in longitudinal cohort studies have also demonstrated that residual selection bias and confounding can render standard adjustment and weighting approaches to be potentially insufficient (Nohr & Liew, 2018). We extend those approaches here, in cohorts with substantial variation in the level of attrition over historical time, demonstrating that the strength of unobservable associations with losses to follow‐up become principle determinants that threaten inference.

The implications of these results for developmental patterns of substance use across the life course underscore that patterns of adult alcohol consumption can be predicted from adolescent use but that there are many sources of variation that also influence the magnitude of this risk. Indeed, the relationship between adolescent binge drinking and age 29/30 binge drinking is moderate, and varies across birth cohorts. Adolescent binge drinking has been declining for more than 30 years (Miech et al., 2019), and in 2018 was at its lowest level recorded in surveillance studies of adolescents (Miech et al., 2019). Concomitantly, adult binge drinking and other indicators of alcohol consumption are remaining steady or increasing across at least the past decade (Patrick et al., 2019; Schulenberg et al., 2019). Furthermore, there is an inverse correlation by birth cohort in relationship between age 18 alcohol consumption and the growth of consumption through the transition to adulthood (Jager, Keyes, & Schulenberg, 2015; Jager, Schulenberg, O'Malley, & Bachman, 2013), suggesting that although public health success in reducing adolescent drinking has been an achievement, adult drinking continues to increase, and public health efforts aimed at the transition to adulthood should be increased.

There are limitations to generalizability and inference from the present study that should be noted. As in most epidemiological observational cohort studies, assessments are based on self‐report of respondents and include measurement error. The validity of IPW as a source of confounder control depends on proper functional form of included variables and the lack of omitted variables, which cannot be empirically verified. Additionally, the MTF study excludes students who left school prior to the twelfth grade, thus the present results are unlikely to generalize to individuals who did not complete high school.

In summary, we recommend for future work that investigators include bias analysis along with IPW strategies, to determine the sensitivity of results to a range of potential attrition patterns. User‐friendly statistical macros and programs for including bias analysis are available (Lash et al., 2009) and are commensurate with the increase in calls for including assessments of systematic error in research studies along with traditional assessments of random error (VanderWeele & Ding, 2017). Continued attention on public health approaches that reduce harm due to alcohol use by conducting high‐quality longitudinal research across the life course to inform prevention, intervention, and etiologic investigation remains among the most central ways in which alcohol‐related morbidity and mortality can be addressed.

## DECLARATION OF INTEREST STATEMENT

K. Keyes and C. Rutherford report personal fees related to consultation with Plaintiff representatives in ongoing opioid product litigation. Other authors declare no potential conflict of interest and have no financial relationships with commercial interests related to the material in this article.

## Supporting information


**Table S1** Attrition distributions by variables used to calculate a summary inverse probability of attrition weight
Online Table 2. Observed risk of binge drinking at age 29/30 for each quartile of predicted attrition risk and the risk difference of binge drinking at age 29/30 by baseline binge status, stratified by IPW quartile
