# Supplementary material for: When does attrition lead to biased estimates of alcohol consumption? Bias analysis for loss to follow‐up in 30 longitudinal cohorts
Source: Int J Methods Psychiatr Res. 2020 Jul 13;29(4):e1842. doi: 10.1002/mpr.1842 (PMC7723204; doi:10.1002/mpr.1842)
Supplement: Supplementary file 1 — Table S1 Attrition distributions by variables used to calculate a summary inverse probability of attrition weight Online Table 2. Observed risk of binge drinking at age 29/30 for each quartile of predicted attrition risk and the risk difference of binge drinking at age 29/30 by baseline binge status, stratified by IPW quartile [file MPR-29-e1842-s001.docx]

Online Table 1. Attrition distributions by variables used to calculate a summary inverse probability of attrition weight

|  |  | Age 29/30 attrition % | | | | | | |
| --- | --- | --- | --- | --- | --- | --- | --- | --- |
|  | Baseline N | Total | 1976-1980 | 1981-1985 | 1986-1990 | 1991-1995 | 1996-2000 | 2001-2005 |
| Total | 73,298 | 48.45 | 31.14 | 40.65 | 49.46 | 51.61 | 55.92 | 61.33 |
| Binge drinking |  |  |  |  |  |  |  |  |
| Yes | 27,925 | 50.86 | 33.57 | 42.88 | 52.94 | 56.32 | 60.66 | 66.99 |
| No | 41,571 | 45.79 | 27.38 | 37.42 | 45.82 | 48.20 | 52.15 | 57.53 |
| Binge drinking at FU1 |  |  |  |  |  |  |  |  |
| Yes | 20,506 | 34.03 | 23.55 | 31.05 | 36.71 | 38.07 | 40.18 | 42.25 |
| No | 29,231 | 33.78 | 22.34 | 31.15 | 36.82 | 36.08 | 37.70 | 39.74 |
| Binge drinking at FU2 |  |  |  |  |  |  |  |  |
| Yes | 20,635 | 29.11 | 20.44 | 27.61 | 32.30 | 31.63 | 32.14 | 34.83 |
| No | 26,192 | 28.57 | 18.33 | 26.81 | 31.67 | 31.29 | 31.36 | 34.97 |
| Binge drinking at FU3 |  |  |  |  |  |  |  |  |
| Yes | 17,762 | 25.18 | 16.97 | 24.31 | 27.10 | 27.52 | 27.88 | 31.14 |
| No | 26,315 | 22.77 | 15.42 | 22.18 | 25.58 | 24.02 | 24.65 | 27.66 |
| Binge drinking at FU4 |  |  |  |  |  |  |  |  |
| Yes | 14,911 | 20.62 | 14.75 | 20.42 | 21.92 | 23.21 | 21.86 | 24.30 |
| No | 26,219 | 17.93 | 11.71 | 17.60 | 20.82 | 19.09 | 19.27 | 21.96 |
| Binge drinking at FU5 |  |  |  |  |  |  |  |  |
| Yes | 13,048 | 16.08 | 11.81 | 16.39 | 18.02 | 17.39 | 15.48 | 19.35 |
| No | 25,917 | 13.55 | 8.75 | 12.98 | 15.53 | 15.09 | 14.29 | 17.23 |
| Sex |  |  |  |  |  |  |  |  |
| Men | 35,955 | 54.74 | 35.99 | 46.41 | 55.23 | 59.17 | 63.22 | 68.38 |
| Women | 37,337 | 42.39 | 26.30 | 35.13 | 43.83 | 44.32 | 48.80 | 54.90 |
| Race/ethnicity |  |  |  |  |  |  |  |  |
| White | 54,298 | 42.84 | 26.72 | 35.69 | 44.06 | 45.93 | 50.86 | 56.66 |
| Black | 7,785 | 67.77 | 52.57 | 62.15 | 72.76 | 71.67 | 73.28 | 74.43 |
| Hispanic | 5,189 | 65.45 | 52.61 | 57.76 | 63.47 | 65.31 | 66.25 | 72.03 |
| Parent education |  |  |  |  |  |  |  |  |
| College + | 30,471 | 46.23 | 28.95 | 37.68 | 44.91 | 48.18 | 51.92 | 56.73 |
| < College | 40,275 | 49.45 | 31.58 | 41.99 | 51.93 | 53.39 | 59.05 | 64.81 |
| Drug strata weight |  |  |  |  |  |  |  |  |
| 0.33 | 21,949 | 52.74 | 35.84 | 43.29 | 56.50 | 59.51 | 63.68 | 68.07 |
| 1.0 | 51,349 | 46.61 | 28.18 | 39.05 | 47.01 | 49.48 | 52.90 | 58.62 |
|  |  | Mean (SD) | | | | | | |
|  |  | Total | 1976-1980 | 1981-1985 | 1986-1990 | 1991-1995 | 1996-2000 | 2001-2005 |
| GPA |  |  |  |  |  |  |  |  |
| Age 29/30 Attrition |  | 5.50 (1.98) | 5.12 (1.90) | 5.12 (1.91) | 5.23 (1.91) | 5.48 (1.97) | 5.78 (1.98) | 5.93 (1.99) |
| No age 29/30 Attrition |  | 6.20 (1.93) | 5.81 (1.91) | 5.90 (1.91) | 6.05 (1.90) | 6.33 (1.89) | 6.61 (1.89) | 6.88 (1.85) |

Online Table 2. Observed risk of binge drinking at age 29/30 for each quartile of predicted attrition risk and the risk difference of binge drinking at age 29/30 by baseline binge status, stratified by IPW quartile

| Predicted attrition risk* quartile | Minimum | Maximum | Baseline binge drinking | Age 29/30 binge drinking | Risk of age 29/30 binge drinking by IPW quartile | Risk of age 29/30 binge drinking by baseline binge drinking (ref=no baseline binge drinking), **stratified** by IPW quartile |
| --- | --- | --- | --- | --- | --- | --- |
|  |  |  | % | % | RR (95% C.I) | RD (95% C.I) |
| 1 | 0.04 | 0.07 | 19.00 | 12.35 | REF | 0.14 (0.11, 0.17) |
| 2 | 0.07 | 0.08 | 30.13 | 23.35 | 1.12 (1.11, 1.13) | 0.16 (0.14, 0.19) |
| 3 | 0.08 | 0.10 | 43.63 | 34.77 | 1.25 (1.23, 1.27) | 0.24 (0.21, 0.26) |
| 4 | 0.10 | 0.36 | 52.44 | 44.87 | 1.39 (1.36, 1.40) | 0.28 (0.25, 0.31) |

Note: *prediction model accuracy (area under the curve) = 0.59
